# Supplementary material for: Clustering of circular consensus sequences: accurate error correction and assembly of single molecule real-time reads from multiplexed amplicon libraries
Source: BMC Bioinformatics. 2018 Aug 20;19:302. doi: 10.1186/s12859-018-2293-0 (PMC6102811; doi:10.1186/s12859-018-2293-0)
Supplement: Supplementary file 1 — Table S1. PacBio reads of insert protocol output metrics. Table S2. Padded and barcoded primer sequences used for amplification of six maize lines. Figure S1. Dot-plots of alignments between amplicon reference sequences and inaccurate consensus sequences generated by LAA. (PDF 321 kb) [file 12859_2018_2293_MOESM1_ESM.pdf]

Clustering of Circular Consensus Sequences: Accurate Error Correction and Assembly of Single Molecule Real-Time Reads from Multiplexed Amplicon Libraries

Felix Francis, Michael D. Dumas, Scott B. Davis and Randall J. Wisser

Supplementary Tables

Table S1. **PacBio reads of insert protocol output metrics.**

Metric	Amplicon library	
	Single	Multiplex
Reads of Insert	136,008,130	210,038,102
Mean Read Length of Insert	3,697	4,094
Mean Read Quality of Insert	98.54%	98.39%
Mean Number of Passes	9.39	9.00

Table S2. **Padded and barcoded primer sequences used for amplification of six maize lines.**

Sample	Primer	Sequence
P39	11.4_f_1	GTTAGCTATACATGACTCTGCGCGGCTGTGAAGATGTCATGGACA
Tx303	11.4_f_2	GTTAGTGTGTATCAGTACATGGCGGCTGTGAAGATGTCATGGACA
Mo17	11.4_f_3	GTTAGTATGTGATCGTCTCTCGCGGCTGTGAAGATGTCATGGACA
Hp301	11.4_f_4	GTTAGTACGACTACATATCAGGCGGCTGTGAAGATGTCATGGACA
B73	11.4_f_5	GTTAGTATCTCTGTAGAGTCTGCGGCTGTGAAGATGTCATGGACA
CML277	11.4_f_6	GTTAGTCTATGTCTCAGTAGTGCGGCTGTGAAGATGTCATGGACA
P39	11.4_r_1	GTTAGGCAGAGTCATGTATAGTGGAACAAGAGCTGGCCTGTTCGA
Tx303	11.4_r_2	GTTAGCATGTACTGATACACATGGAACAAGAGCTGGCCTGTTCGA
Mo17	11.4_r_3	GTTAGGAGAGACGATCACATATGGAACAAGAGCTGGCCTGTTCGA
Hp301	11.4_r_4	GTTAGCTGATATGTAGTCGTATGGAACAAGAGCTGGCCTGTTCGA
B73	11.4_r_5	GTTAGAGACTCTACAGAGATATGGAACAAGAGCTGGCCTGTTCGA
CML277	11.4_r_6	GTTAGACTACTGAGACATAGATGGAACAAGAGCTGGCCTGTTCGA
P39	2.2_f_1	GTTAGCTATACATGACTCTGCGCGGCATACAACAGGCAAAGTTAGC
Tx303	2.2_f_2	GTTAGTGTGTATCAGTACATGGCGGCATACAACAGGCAAAGTTAGC
Mo17	2.2_f_3	GTTAGTATGTGATCGTCTCTCGCGGCATACAACAGGCAAAGTTAGC
Hp301	2.2_f_4	GTTAGTACGACTACATATCAGGCGGCATACAACAGGCAAAGTTAGC
B73	2.2_f_5	GTTAGTATCTCTGTAGAGTCTGCGGCATACAACAGGCAAAGTTAGC
CML277	2.2_f_6	GTTAGTCTATGTCTCAGTAGTGCGGCATACAACAGGCAAAGTTAGC
P39	2.2_r_1	GTTAGGCAGAGTCATGTATAGGGCTGCTATGGTCTGTATCGTCTCCAAC
Tx303	2.2_r_2	GTTAGCATGTACTGATACACAGGCTGCTATGGTCTGTATCGTCTCCAAC
Mo17	2.2_r_3	GTTAGGAGAGACGATCACATAGGCTGCTATGGTCTGTATCGTCTCCAAC
Hp301	2.2_r_4	GTTAGCTGATATGTAGTCGTAGGCTGCTATGGTCTGTATCGTCTCCAAC

B73	2.2_r_5	GTTAGAGACTCTACAGAGATAGGCTGCTATGGTCTGTATCGTCTCCAAC
CML277	2.2_r_6	GTTAGACTACTGAGACATAGAGGCTGCTATGGTCTGTATCGTCTCCAAC
P39	2.1_f_1	GTTAGCTATACATGACTCTGCTTGTGGCATCAGTAGGGTCTGAAAC
Tx303	2.1_f_2	GTTAGTGTGTATCAGTACATGTTGTGGCATCAGTAGGGTCTGAAAC
Mo17	2.1_f_3	GTTAGTATGTGATCGTCTCTCTTGTGGCATCAGTAGGGTCTGAAAC
Hp301	2.1_f_4	GTTAGTACGACTACATATCAGTTGTGGCATCAGTAGGGTCTGAAAC
B73	2.1_f_5	GTTAGTATCTCTGTAGAGTCTTTGTGGCATCAGTAGGGTCTGAAAC
CML277	2.1_f_6	GTTAGTCTATGTCTCAGTAGTTTGTGGCATCAGTAGGGTCTGAAAC
P39	2.1_r_1	GTTAGGCAGAGTCATGTATAGCCTTCCCTTAACCAAAGTTGAATAGCAG
Tx303	2.1_r_2	GTTAGCATGTACTGATACACACCTTCCCTTAACCAAAGTTGAATAGCAG
Mo17	2.1_r_3	GTTAGGAGAGACGATCACATACCTTCCCTTAACCAAAGTTGAATAGCAG
Hp301	2.1_r_4	GTTAGCTGATATGTAGTCGTACCTTCCCTTAACCAAAGTTGAATAGCAG
B73	2.1_r_5	GTTAGAGACTCTACAGAGATACCTTCCCTTAACCAAAGTTGAATAGCAG
CML277	2.1_r_6	GTTAGACTACTGAGACATAGACCTTCCCTTAACCAAAGTTGAATAGCAG
P39	13.4_f_1	GTTAGCTATACATGACTCTGCTCCTCATTCTCCTCTCGGAATCGCT
Tx303	13.4_f_2	GTTAGTGTGTATCAGTACATGTCCTCATTCTCCTCTCGGAATCGCT
Mo17	13.4_f_3	GTTAGTATGTGATCGTCTCTCTCCTCATTCTCCTCTCGGAATCGCT
Hp301	13.4_f_4	GTTAGTACGACTACATATCAGTCCTCATTCTCCTCTCGGAATCGCT
B73	13.4_f_5	GTTAGTATCTCTGTAGAGTCTTCCTCATTCTCCTCTCGGAATCGCT
CML277	13.4_f_6	GTTAGTCTATGTCTCAGTAGTTCCTCATTCTCCTCTCGGAATCGCT
P39	13.4_r_1	GTTAGGCAGAGTCATGTATAGATACCACCTCAAGAAAGCAGGCCTA
Tx303	13.4_r_2	GTTAGCATGTACTGATACACAATACCACCTCAAGAAAGCAGGCCTA
Mo17	13.4_r_3	GTTAGGAGAGACGATCACATAATACCACCTCAAGAAAGCAGGCCTA
Hp301	13.4_r_4	GTTAGCTGATATGTAGTCGTAATACCACCTCAAGAAAGCAGGCCTA
B73	13.4_r_5	GTTAGAGACTCTACAGAGATAATACCACCTCAAGAAAGCAGGCCTA
CML277	13.4_r_6	GTTAGACTACTGAGACATAGAATACCACCTCAAGAAAGCAGGCCTA
P39	6.5_f_1	GTTAGCTATACATGACTCTGCCTTGCCGCTAAACCATCTCGCGATT
Tx303	6.5_f_2	GTTAGTGTGTATCAGTACATGCTTGCCGCTAAACCATCTCGCGATT
Mo17	6.5_f_3	GTTAGTATGTGATCGTCTCTCCTTGCCGCTAAACCATCTCGCGATT
Hp301	6.5_f_4	GTTAGTACGACTACATATCAGCTTGCCGCTAAACCATCTCGCGATT
B73	6.5_f_5	GTTAGTATCTCTGTAGAGTCTCTTGCCGCTAAACCATCTCGCGATT
CML277	6.5_f_6	GTTAGTCTATGTCTCAGTAGTCTTGCCGCTAAACCATCTCGCGATT
P39	6.5_r_1	GTTAGGCAGAGTCATGTATAGTTCTTGCGCCCACCTCCTTCTGAAA
Tx303	6.5_r_2	GTTAGCATGTACTGATACACATTCTTGCGCCCACCTCCTTCTGAAA
Mo17	6.5_r_3	GTTAGGAGAGACGATCACATATTCTTGCGCCCACCTCCTTCTGAAA
Hp301	6.5_r_4	GTTAGCTGATATGTAGTCGTATTCTTGCGCCCACCTCCTTCTGAAA
B73	6.5_r_5	GTTAGAGACTCTACAGAGATATTCTTGCGCCCACCTCCTTCTGAAA
CML277	6.5_r_6	GTTAGACTACTGAGACATAGATTCTTGCGCCCACCTCCTTCTGAAA
P39	1.6_f_1	GTTAGCTATACATGACTCTGCAAGCTCTAAGCGCCGCCATGGTTA
Tx303	1.6_f_2	GTTAGTGTGTATCAGTACATGAAGCTCTAAGCGCCGCCATGGTTA
Mo17	1.6_f_3	GTTAGTATGTGATCGTCTCTCAAGCTCTAAGCGCCGCCATGGTTA
Hp301	1.6_f_4	GTTAGTACGACTACATATCAGAAGCTCTAAGCGCCGCCATGGTTA
B73	1.6_f_5	GTTAGTATCTCTGTAGAGTCTAAGCTCTAAGCGCCGCCATGGTTA
CML277	1.6_f_6	GTTAGTCTATGTCTCAGTAGTAAGCTCTAAGCGCCGCCATGGTTA
P39	1.6_r_1	GTTAGGCAGAGTCATGTATAGGCATCTTGGCGTACACCTTGTTGG
Tx303	1.6_r_2	GTTAGCATGTACTGATACACAGCATCTTGGCGTACACCTTGTTGG
Mo17	1.6_r_3	GTTAGGAGAGACGATCACATAGCATCTTGGCGTACACCTTGTTGG
Hp301	1.6_r_4	GTTAGCTGATATGTAGTCGTAGCATCTTGGCGTACACCTTGTTGG
B73	1.6_r_5	GTTAGAGACTCTACAGAGATAGCATCTTGGCGTACACCTTGTTGG
CML277	1.6_r_6	GTTAGACTACTGAGACATAGAGCATCTTGGCGTACACCTTGTTGG

Supplementary Figures

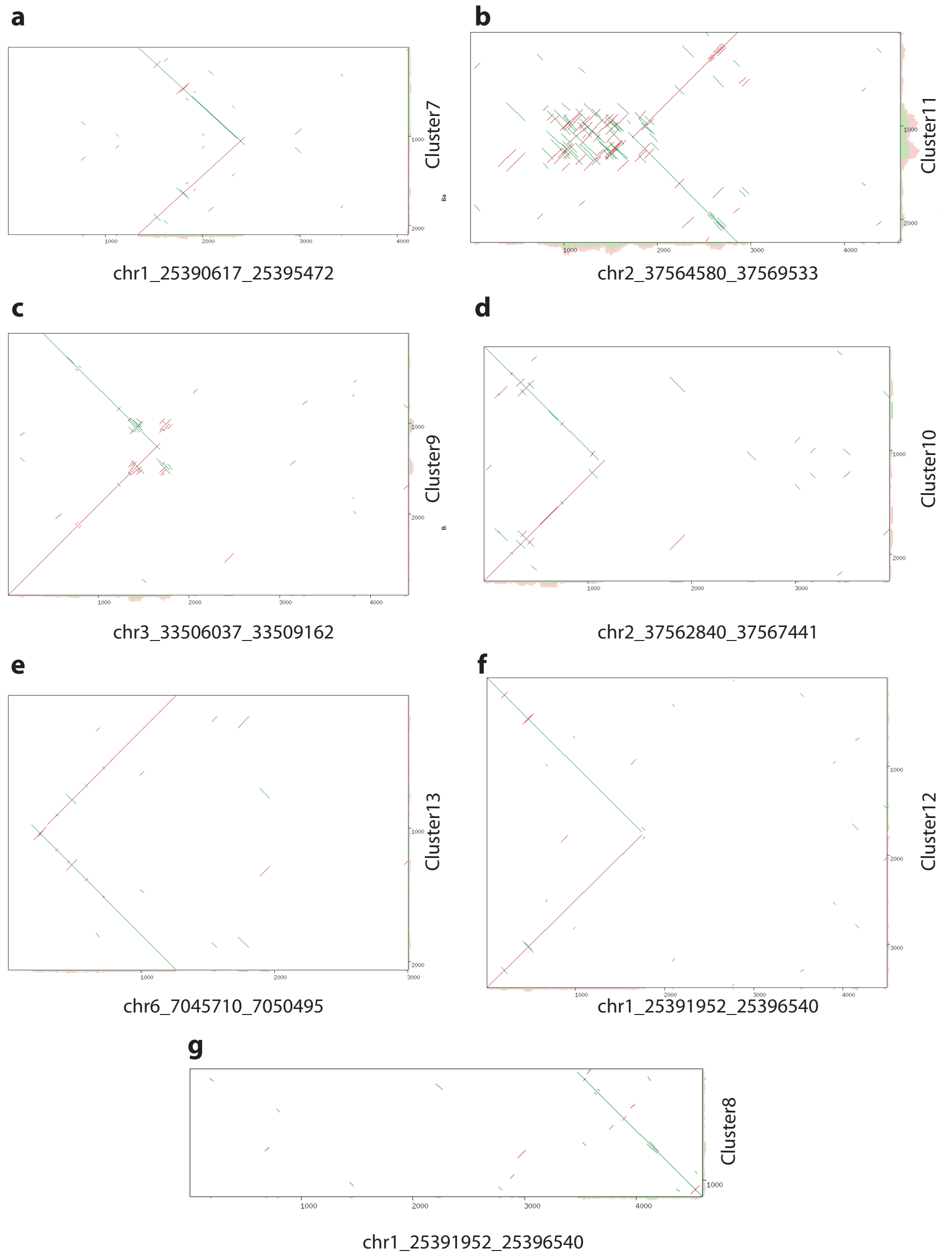

Figure S1. Dot-plots of alignments between amplicon reference sequences and inaccurate consensus sequences generated by LAA. (a-g) The reference sequence for each amplicon is labeled according the chromosome, start and stop position in the reference genome of B73 v3 (x-axes). The consensus sequences are labeled 7-13 (y-axes). Green lines show alignments in the same orientation while red lines show alignments in reverse orientation.
